# Different Gut Microbiomes of Developmental Stages of Field-Collected Native and Invasive Western Bean Cutworm, *Striacosta albicosta*, in Western Nebraska

**DOI:** 10.3390/microorganisms10091828

**Published:** 2022-09-14

**Authors:** Paul A. Ayayee, Austin Currie, Julie A. Peterson

**Affiliations:** 1 Department of Biology, University of Nebraska at Omaha, Omaha, NE 68182, USA; 2 Department of Entomology, West Central Research & Extension Center, University of Nebraska-Lincoln, North Platte, NE 68588, USA

**Keywords:** gut microbiome, stage-specific, western bean cutworm, *S. albicosta*, corn, pest control

## Abstract

While insects harbor gut microbial associates that perform various functions for the host, lepidopterans have not been considered as prime examples of having such relationships. The western bean cutworm, *Striacosta albicosta* (Lepidoptera: Noctuidae), is native to North America and has historically been a significant corn pest in its western distribution. It is currently expanding eastwards and is invasive in these new regions. Using 16S rRNA gene sequencing data, this study focused on characterizing the microbiota associated with field-collected eggs, larvae, adults, and host plant materials of *S. albicosta* in its native range. The diversity of microbiomes varied significantly among *S. albicosta* eggs, larvae, adults, and the host plant materials. Microbial diversity was highest in adult stages relative to other insect stages. Furthermore, *S. albicosta* eggs, larvae, and adults harbored very distinct microbial communities, indicative of stage-specific microbiomes possibly performing different functions. Bacterial taxa underscoring these differences in composition identified four phyla and thirty families across samples. Members of the Firmicutes (Unassigned Lactobacillales), Proteobacteria (*Pseudomonadaceae* and *Moraxellaceae*), Bacteroidota (*Weeksellaceae*), and Chloroflexi dominated across all developmental stages. In addition, cellulose-degrading Lactobacillales (phylum: Firmicutes) dominated larval microbiomes, indicative of larval plant diet. This taxon was comparatively negligible in eggs and adults. Members of Proteobacteria dominated egg and host leaf microbiomes, while members of Bacteroidota dominated nectar-feeding adult gut microbiomes. Our results suggest a possible diet-dependent stage-specific microbiome composition and the potential for using stage-specific microbes as potential biological control tools against this important pest moving forward.

## 1. Introduction

The past two decades have seen a tremendous increase in the availability of data detailing the microbial compositions of the gut microbiomes of various insects and the postulated functions of these gut microbiomes [1,2]. In some particular instances, the functions of insect gut microbiomes have been demonstrated to include nitrogen provisioning [3], essential amino acid provisioning [4,5,6,7], and an overall propensity for essential nutrient provisioning followed by detoxification and digestion [8]. These functions have been uncovered in both insect species considered pests and non-pests.

The western bean cutworm, *Striacosta albicosta* (Smith) (Lepidoptera: Noctuidae), feeds primarily on corn and dry beans and is native to western North America. It is an economically damaging pest in parts of its native range, particularly in western Nebraska, Kansas, and eastern Colorado [9,10,11]. However, its range has expanded eastwards in continental North America since approximately 2000 and was most recently detected in Prince Edward Island, Canada, in 2017 [12], and Quebec, Canada, in 2020 [13]. *S. albicosta* is now considered a primary pest of corn in Ontario, Canada, and U.S. states in the Great Lakes Region [14]. Larvae hatch from eggs laid on host plant leaves in approximately 5–7 day following oviposition, proceed to feed on the chorion before switching to feeding on the reproductive parts of the host plant (first tassels and pollen, followed by silks and developing ear kernels), then drop to the ground around the sixth larval instar, where they burrow into the soil to overwinter as prepupae [12,15]. The eastward expansion of this insect pest has not been definitively associated with host range expansion or host shifts. Corn and dry beans are still the preferred host plants in the newly expanded ranges. However, the insect utilizes some secondary host plants for short periods, but larval development and survival are suboptimal [12].

The potential influences of the gut microbiome on *S. albicosta*’s ecology (host shifts and range expansion) is difficult to ascertain, given the scarcity of data regarding the composition and functions of the gut microbiome of lepidopterans in general [16]. However, a few exceptions include *Spodoptera littoralis* [17,18], the velvet bean caterpillar [19], the domestic silkworm [20,21], the neotropical butterfly *Heliconius erato* [22], and the European corn borer [23]. Studies investigating gut microbiome composition and functions of lepidopteran gut microbiomes enhance our fundamental understanding of these associations in this order and of insects in general. In addition, these studies illuminate our understanding of how gut microbes may be involved in the range expansions, host shifts, and potential novel pest management strategies of major lepidopteran agricultural pests.

These questions are relevant in the case of *S. albicosta*, an insect that recently expanded eastwards from its native western range. Herein, we utilized high-throughput sequencing of DNA extracted from host plants (corn), eggs, larvae, and adults of the western bean cutworm in its native range and characterized the gut microbiome to (1) assess the gut microbial composition among the developmental stages of the insect and (2) determine which bacterial taxa are shared across developmental stages or are characteristic of a particular developmental stage. This is the first step in investigating this pest’s biological control through manipulating bacteriophages associated with stage-specific bacteria in its gut microbiomes.

## 2. Materials and Methods

### 2.1. Sample Collection

We collected western bean cutworm (*S. albicosta*) egg masses, early instar larvae (1st through 3rd instar), and adults in cornfields at four different sites in west central Nebraska (Kearney: 40.759310°, −99.138466°, North Platte: 41.085397°, −100.773569°, Brule: 41.158948°, −102.026904°, and Grant: 40.857193°, −101.699452°) in mid to late July 2021. Larval instars represented the stages that are targeted by insecticide applications in the field, as the first three instars feed at exposed sites on the tassel and corn plant, prior to entering the ears, where later instars are no longer accessible to insecticide applications. The four sampling sites are located within the native western range of this species, where corn (particularly, non-Bt strain corn) is the preferred and dominant host plant. Samples were collected from monitoring and trapping sites under the management of UNL’s Agroecosystems Entomology Lab [24,25,26]. Collected samples were stored on ice in the field and frozen at −20 °C upon return to the laboratory until DNA extraction.

### 2.2. Sample Processing, DNA Extraction, and Illumina Sequencing

Before DNA extraction, we surface-sterilized egg masses (n = 7, pool of 10 individual eggs), larvae (n = 8, pool of 2–3 larvae), and plant materials (n = 3) by washing in a 1% detergent solution for 1 min, followed by two one-minute rinses in deionized (DI) water. We similarly surface-sterilized adults (n = 9 individual adults) before DNA extraction, after the removal of wings and legs. We performed DNA extraction using the FastDNA SPIN Kit for Soil (MP Biomedicals, Irvine, CA, USA) according to the manufacturer’s directions. We verified the presence of the microbial 16S rRNA marker gene in all extracted DNA samples via PCR using the universal 27F and 1492 R bacterial primer pair [27]. Samples were submitted for high-throughput paired-end Miseq library preparation and sequencing at the University of Nebraska Medical Center Genomics Core. Briefly, a limited-cycle PCR reaction was performed on each sample to create a single amplicon, including the V4 (515-F) and V5 (907-R) variable regions [28]. The resulting libraries were validated using the Agilent BioAnalyzer 2100 DNA 1000 chip (Agilent, Santa Clara, CA, USA), and DNA was quantified using the Qubit 3.0 (Qubit^TM^, Thermofisher, Waltham, MA, USA). A pool of the libraries was loaded into the Illumina MiSeq at 10 pM (pico molar). The pool was spiked with 25% PhiX (a bacteriophage) at 10 pM for MiSeq run quality as an internal control [29] to generate 300 bp paired ends with the 600 cycle kit (version 3). The raw reads were deposited into the Sequence Read Archive database (Accession number: PRJNA821530).

### 2.3. Processing Data and Statistical Analyses

Acquired fastq primer-trimmed Miseq paired-end reads from the sequencing center were processed using DADA2 [30]. Across both forward and reverse reads, filtering excluded reads with more than two expected erroneous base calls, any reads identified as part of the PhiX bacteriophage genome for quality control, and reads with less than 175 base pairs. Forward reads were truncated to 250 base pairs and reverse reads to 200 base pairs. Truncation was done to maintain median quality scores above 30 across samples. Reads were merged, and chimeras were subsequently filtered out. We determined amplicon sequence variants (ASVs) and representative sequences against the SILVA 138.1 16S rRNA gene reference database [31]. We combined the count and taxonomy information for the generated ASVs into a classical ASV table, and further analyses were carried out in QIIME v.1.8 [32,33]. Before analyses, we curated the table by removing any reads unclassified at the bacterial or archaeal domain level. In addition, we removed all reads assigned as chloroplast at the order level before analyses, since they were the most due plant contaminant in the insect and leaf samples. Finally, samples with less than 1000 reads per sample were removed from the table before analyses. We then summarized the filtered and curated ASV table to the family level, and all subsequent analyses were carried out on this table.

Briefly, we rarefied the family-level table to 1060 reads per sample and replicated ten times across all samples. The rationale and justification for rarefying have been discussed here [34,35]. For alpha diversity, the diversity matrices chao1 [36], Simpson’s index [37], Shannon’s evenness [38], and observed_otus were calculated in QIIME, and significant differences among categorical groupings were determined via non-parametric Wilcoxon tests in JMP Pro 15 (S.A.S., Cary, NC, USA). We generated the Bray-Curtis dissimilarity distance matrix [39] using the 1060-rarefied table. The distance matrix was used to calculate the non-metric multidimensional scales (NMDS) in QIIME. The NMDS scales are used to visualize categorical sample groupings that differ in microbiome composition following a test of differences among these categorical groupings via a permutational multivariate analysis of variance (PERMANOVA) [40] in QIIME using the compare_categories.py command.

## 3. Results

Overall, we obtained ~2.5 million reads from the sequencing effort. Filtering, merging, chimera removal, and further curating of the resulting combined count and taxonomy table (removal of unassigned reads at the domain level and samples with fewer than 1000 reads per sample) resulted in ~23% of retained reads (577,591 reads) distributed across 21 samples [eggs (n = 6), larvae (n = 4), adults (n = 8), and leaves (n = 3)], yielding 1044 ASVs (mean reads per sample = 9152.818; Minimum: 1019.000, Maximum: 51,538.000). Rarefaction curves for the species’ diversity and richness indices at ~1000 reads per sample indicate that most microbial diversity had been sufficiently sampled across samples (Figure S1).

**Figure 1:**
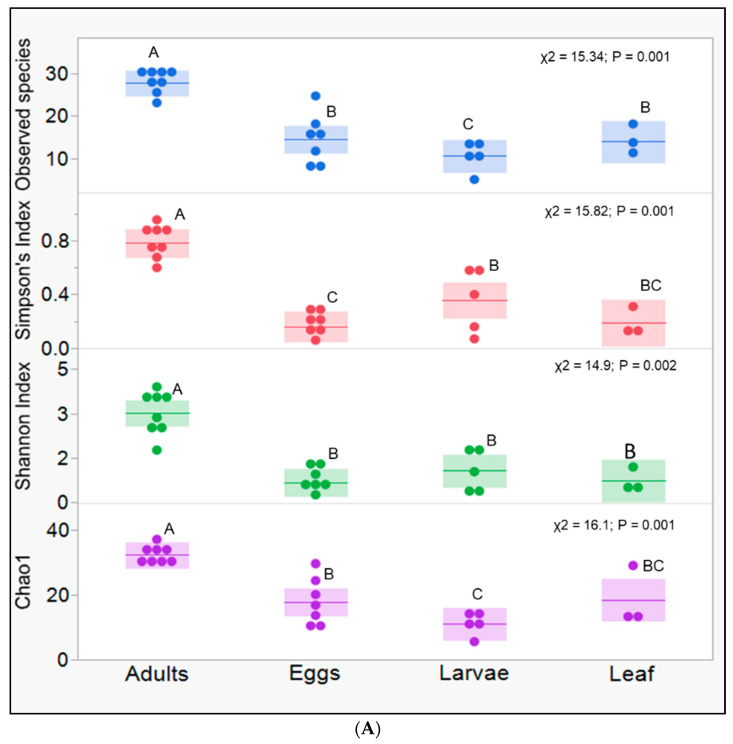

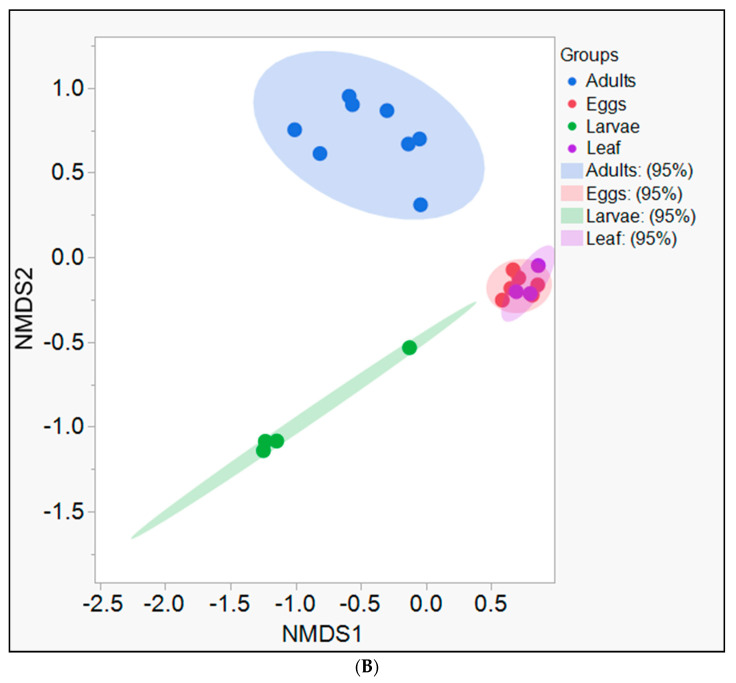
Microbiome dynamics vary across developmental stages (egg, larvae, and adults) of field-collected western bean cutworm *Striacosta albicosta* and its dietary material. (**A**) Boxplots of microbial species’ diversity and richness indices evaluated across developmental stages in this study. (**B**) Non-metric multidimensional scaling plot showing variations in microbiome composition among WBC developmental stages and its dietary material. The colors in panels A and B are not intended to mean the same things.

**Figure 2:**
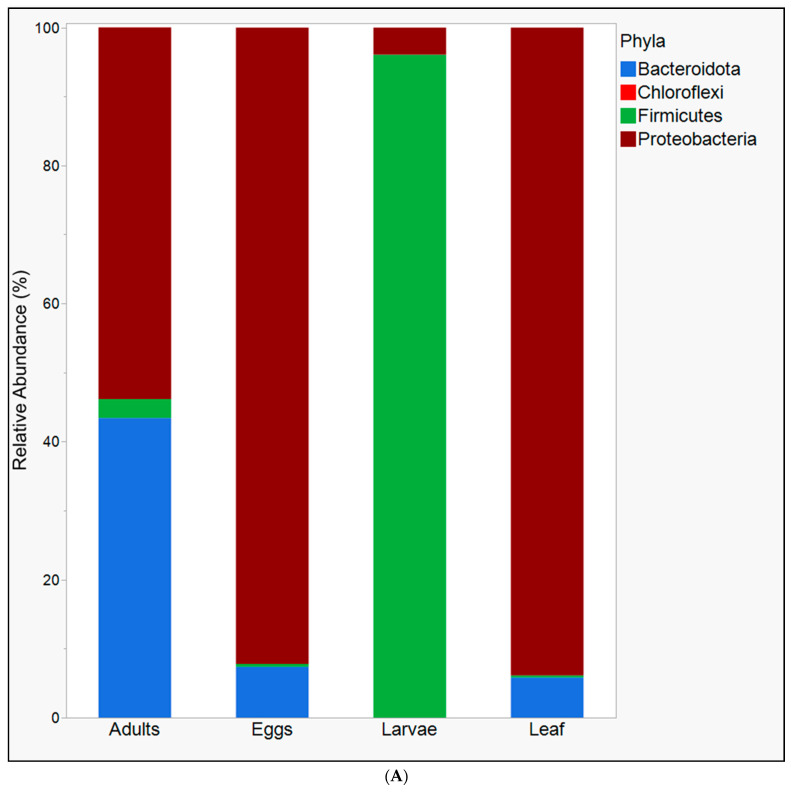

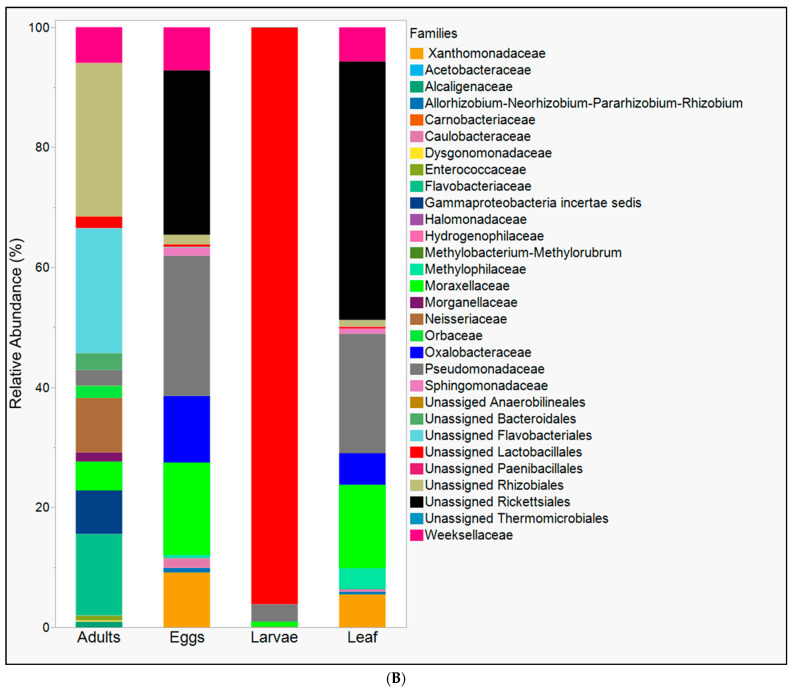
Microbiota differences across field-collected western bean cutworm *Striacosta albicosta* developmental stages and its dietary material. Differentially abundant microbial (**A**) phyla and (**B**) families (*p* < 0.05) across developmental stages.

This study uncovered significant microbiome diversity and composition differences across the three (eggs, larvae, and adult) field-collected developmental stages of *S. albicosta* and host leaves. We removed chloroplast reads from consideration to avoid overestimating bacterial diversity in the gut of field-collected *S. albicosta* and leaf samples. Overall, the microbial community diversity among insect samples was significantly higher in adults compared to eggs and larvae for chao1 (Figure 1A). The remaining three diversity indices were comparable for adults and eggs. Still, both were significantly higher than the larval diversity (Figure 1A). The diversity in leaf samples also tended to be higher than the larval diversity but was comparable to the calculated diversity in adults and eggs (Figure 1A). The differences in the alpha diversity indices among sample groups were underscored by significant differences in the microbiome community composition among eggs, larvae, adults, and plant leaves in the NMDS plot generated using the Bray-Curtis dissimilarity matrix (Figure 1B; NMDS stress = 0.06; PERMANOVA: test-statistic = 6.16, *p* < 0.001). Clustered samples (eggs, leaves) are distinct from adults and larvae, which are further clustered separately from each other (Figure 1B). Thirty-two bacterial families (summarized across four bacterial phyla) were differentially abundant across sample groups and, thus, responsible for the observed differences in the community composition (Figure 2A,B). Unassigned Rickettsiales, Xanthomonadaceae, *Pseudomonadaceae*, *Moraxellaceae* (all Proteobacteria phyla), and *Weeksellaceae* (Bacteroidata phylum) were significantly more abundant in eggs and leaves relative to adults, with Oxalobacteraceae, Sphingomonadaceae, and the *Rhizobium* complex (*Allorhizobium-Neorhizobium-Pararhizobium-Rhizobium*) being more abundant in eggs relative to leaf samples (Figure 2B). Unassigned Lactobacillales (Firmicutes phylum) was disproportionately abundant in larvae (~96.1%) compared to any other sample group. In adults, unassigned *Flavobacteriales*, *Flavobacteriaceae*, *Weeksellaceae* (Bacteroidota phylum), unassigned Rhizobiales, Neisseriaceae, and Gammaproteobacteria incertae sedis (Proteobacteria phyla) were significantly more abundant relative to other sample groups (Figure 2B). *Pseudomonadaceae* was the prominent differentially abundant bacterial family across all sample groups. These families were distributed across four main phyla (Figure 2A).

## 4. Discussion

While most insects are known to harbor complex gut microbial associates that perform various functions for the host, lepidopterans have not been considered prime examples of such associations. In this study, we uncovered significant differences in microbiome diversity and community composition among field-collected eggs, larvae, and adult stages of the western bean cutworm, *S. albicosta,* and its major host plant, corn (*Zea mays*)*,* in west central Nebraska. This study is the first to characterize the gut microbiome of various developmental stages of field-collected *S. albicosta* in its native western range in North America, suggesting that, perhaps, there exist stage-specific residential gut microbiomes in this species. The results presented and discussed herein add to a small but growing body of work detailing the composition (and similarities) of the gut microbiomes of various lepidopterans relative to other insect orders [16]. The results also provide insights into which gut microbial members are dominant across the various developmental stages and may be potential targets for manipulation via biological control within an integrated pest management framework.

Across *S. albicosta* developmental stages, we determined the highest species richness and community diversity to be in adults (nectar-feeding), followed by eggs, and the lowest to be in larvae. This trend is consistent with previous studies that analyzed the gut microbiome of Lepidoptera through NGS techniques in Egyptian cotton leafworm [18], European corn borer [23], the velvet bean caterpillar [19], domestic silkworm [20,21], and the Red postman [22], and, to a lesser extent, via cloning and sanger sequencing techniques in Egyptian cotton leafworm and cotton bollworm [17]. In all these studies, as well as this study, most of the microbial diversity could be summarized into three significant phyla: Bacteroidetes, Firmicutes, and Proteobacteria, and to a lesser extent, Chloroflexi. This is unclear, but it is possible to infer that the larvae, upon hatching from eggs and switching to a foliar diet, acquired gut microbiota through screening processes that resulted in the dominance of functionally relevant taxa. Functionally relevant taxa in Lepidoptera may include lignocellulose-degrading bacteria, such as *Enterococcus* sp. (Firmicutes phylum) and members of the Enterobacteriaceae (Proteobacteriaceae phylum) [41]. The comparatively alkaline pH in the gut of lepidopterans compared to other insect orders [42,43] and the carbon-rich diet may have selected this relatively low-diversity but functionally relevant gut microbial community in members of this order [41]. This selective process is evident in the dominance of unassigned Lactobacillales (phylum Firmicutes) in larval gut microbiomes compared to eggs and adult stages. It could be surmised that the dominance of lactic acid bacteria in the larvae may be relevant for degrading the carbohydrate-rich diet of *S. albicosta* larvae, as has been determined in a variety of other lepidopteran larvae [18,41,44,45]. Finally, members of the bacterial order Pseudomonadales (*Pseudomonadaceae* and *Moraxellaceae*; class Gammaproteobacteria) were also abundant in eggs and adults and to a lesser extent in larvae. Several bacterial orders and families under the class gammaproteobacteria are recognized members of the gut microbiome across several insect species, serving as providers of metabolic nitrogen [3,46,47], essential amino acids [4,44], and microbe-derived hydrolytic enzymes [45,46]. These representative taxa may be performing similar functions in *S. albicosta*. The dominance of members of the Lactobacillales in *S. albicosta* larvae and their relative absence in the leaf samples potentially highlights the need to re-examine the claim that lepidopteran caterpillars do not have a gut microbiome nor need one to be successful herbivores [47], and the need to factor in differences in gut-associated microbiota between lab-read and field-collected lepidopterans in experimental studies and potential biotechnological applications.

One potential biotechnological application of possible interest may include utilizing the members of the WBC gut microbiome as biological control agents. For example, about four microbial taxa, unassigned Lactobacillales, *Pseudomonadaceae*, *Moraxellaceae*, and *Weeksellaceae*, were detected across *S. albicosta* developmental stages (more abundant in eggs and larvae compared to adults). The presence of these taxa across these various stages suggests the persistence of these taxa in this holometabolous insect. This could have implications for using these taxa as potential non-pathogenic microbial biological control agents against WBC. Although ~99% of most members of insect gut microbiomes are not easily cultivable, the relatively sparse gut microbiome of *S. albicosta* and the dominance of lactic acid bacteria (LAB) in larvae may provide a window of opportunity. When provided with facultative to obligate anaerobic conditions, sugar and carbohydrate sources, and more extended incubation periods on selective media, many LABs are successfully isolated from environmental samples using traditional microbiological techniques [48,49].

Furthermore, the adoption and modification of emerging cultivation techniques that make it easy and feasible to enrich Firmicutes (such as lactic acid bacteria) could also be utilized to culture these members of the *S. albicosta* gut microbiome [50]. Firmicute-specific bacteriophages have been used to limit biofilm production by unwanted bacteria on food production surfaces and extend products’ shelf lives by targeting bacteria that cause food spoilage [51,52,53]. Given this extensive usage, it is possible to modify and adapt the knowledge and insights from the food and dairy industry to bacterial host cell recognition and infection, phage propagation within the bacterial host cell, phage specificity, and biological assays in the *S. albicosta* system. Thus, for example, representative LAB isolates from *S. albicosta* larvae could be used as vehicles for altered bacteriophages isolated from *S. albicosta* larvae to disrupt the larval gut microbiome and interfere with insect physiology and development (and thus the inability of the larvae to digest plant-based diet efficiently, leading to increased mortality, decreased pupation and eclosion, and reduced pest incidences). In addition, these technologies have potential biological control applications, as has been demonstrated in the food and dairy industries [53]. Presently, protocols for rearing *S. albicosta* under laboratory conditions have been developed [26] and are being optimized for the increased fecundity and survival of laboratory-reared *S. albicosta* in order to assess potential control strategies.

In conclusion, the results presented in this study demonstrate that: (1) For the western bean cutworm, the eggs, larvae, and adult developmental stages harbor distinct gut microbiota. This may be diet-driven, as each stage feeds on distinct dietary materials. (2) Despite the distinct gut microbial assemblages, some bacterial taxa are present (to a varying degree) across the three developmental stages studied. An inference from this result is that these bacterial taxa may represent *S. albicosta*’s potential microbial biological control agents. We provide the first step towards an understanding of the dynamics structuring the composition of the gut microbiota of the holometabolous lepidopteran insect pest and how they may contribute to host ecology.

## Supplementary Materials

The following supporting information can be downloaded at: https://www.mdpi.com/article/10.3390/microorganisms10091828/s1. Figure S1: Microbiome diversity sampling across developmental stages (egg, larvae, and adults) of field-collected western bean cutworm *Striacosta albicosta* and its dietary material. Rarefaction curves near saturation suggests complete sampling across samples.
