# Supplementary material for: Different Gut Microbiomes of Developmental Stages of Field-Collected Native and Invasive Western Bean Cutworm, Striacosta albicosta, in Western Nebraska
Source: Microorganisms. 2022 Sep 14;10(9):1828. doi: 10.3390/microorganisms10091828 (PMC9505167; doi:10.3390/microorganisms10091828)

Supplementary Figure S1: Microbiome diversity sampling across developmental stages (egg, larvae, and adults) of field-collected western bean cutworm *Striacosta albicosta* and its dietary material. Rarefaction curves near saturation suggests complete sampling across samples.

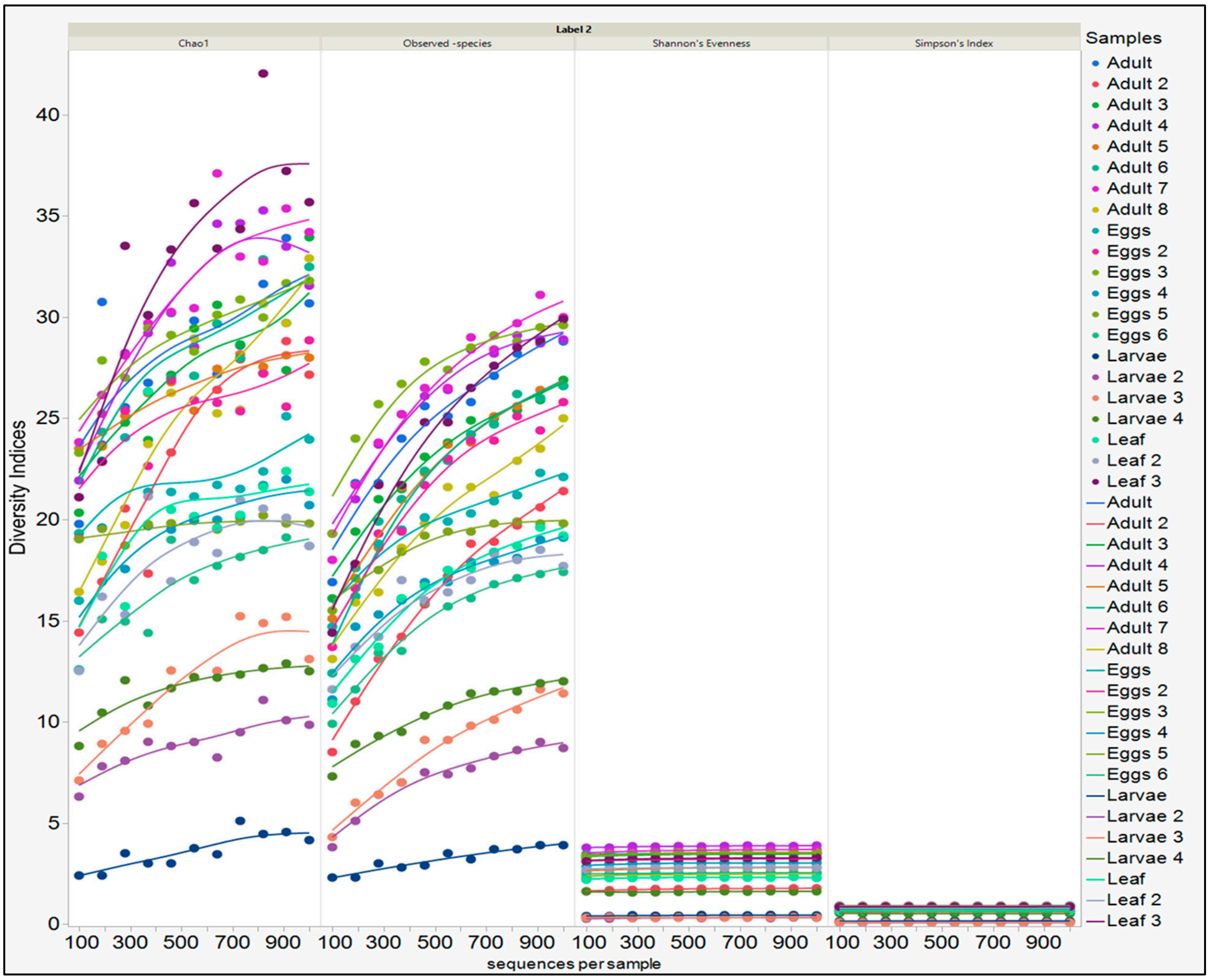

Supplement: Supplementary file 1 [file microorganisms-10-01828-s001.zip › microorganisms-1901649-supplementary.pdf]
